# Neoadjuvant chemoradiotherapy for resectable gastric cancer: A meta-analysis

**DOI:** 10.3389/fonc.2022.927119

**Published:** 2022-08-05

**Authors:** Jiuzhou Chen, Yaru Guo, Miao Fang, Yan Yuan, Youqi Zhu, Yong Xin, Longzhen Zhang

**Affiliations:** ^1^ Department of Radiation, The Affiliated Hospital of Xuzhou Medical University, Jiangsu, China; ^2^ Department of Cancer Institute, Xuzhou Medical University, Jiangsu, China

**Keywords:** resectable gastric cancer, gastrointestinal cancers, neoadjuvant chemoradiotherapy, neoadjuvant chemotherapy, meta-analysis

## Abstract

**Objectives:**

To evaluate the clinical curative effects and toxicity of neoadjuvant chemoradiotherapy for resectable gastric cancer compared to those of neoadjuvant chemotherapy.

**Methods:**

A systematic review and meta-analysis of the randomized controlled trials (RCTs) of neoadjuvant chemoradiotherapy versus neoadjuvant chemotherapy were performed in patients with resectable gastric cancer.

**Results:**

Seven RCTs were included (601 patients; 302 in the neoadjuvant chemoradiotherapy group and 299 in the neoadjuvant chemotherapy group). The neoadjuvant chemoradiotherapy group had an increased number of patients with a complete response [odds ratio (OR) = 3.79, 95% confidence interval (CI): 1.68–8.54, p = 0.001] and improved objective response rate (OR = 2.78, 95% CI: 1.69–4.57, p < 0.0001), 1-year (OR = 3.51, 95% CI: 1.40–8.81, p = 0.007) and 3-year (OR = 2.14, 95% CI: 1.30–3.50, p = 0.003) survival rates, R0 resection rate (OR = 2.21, 95% CI: 1.39–3.50, p = 0.0008), and complete pathologic response (OR = 4.39, 95% CI: 1.59–12.14, p = 0.004). Regarding the incidence of adverse effects after neoadjuvant therapy, only the occurrence rate of gastrointestinal reaction in the neoadjuvant chemoradiotherapy group was higher than that in the neoadjuvant chemotherapy group (OR = 1.76, 95% CI: 1.09–2.85, p = 0.02), and there was no significant difference in other adverse effects. There was no difference in the incidence of postoperative complications between the two groups.

**Conclusion:**

Neoadjuvant chemoradiotherapy for resectable gastric cancer has several advantages in terms of efficacy and safety compared to neoadjuvant chemotherapy. Therefore, neoadjuvant chemoradiotherapy has great potential as an effective therapy for resectable gastric cancers.

**Systematic Review Registration:**

https://inplasy.com/inplasy-2022-3-0164, registration number INPLASY202230164.

## Introduction

Gastric cancer is a malignant tumor with high morbidity and mortality (1). Epidemiological statistics indicate that there were more than one million new cases of gastric cancer and 760,000 deaths in 2020, which rank fifth and fourth, respectively, in the incidence and mortality of cancer worldwide; for patients with advanced gastric cancer, the median survival rate is less than 12 months (2). The incidence is twice as high in men as in women, and the number of new cases continues to increase in younger patients (3). Gastric cancer remains a global health problem.

Surgery is known to play a crucial role in the treatment strategy of gastric cancer, and the prognosis and survival of patients are improved when surgery achieves R0 resection. Preoperative neoadjuvant therapy is the key to achieve R0 resection and has been proven to be effective for potentially resectable gastric cancer (4, 5). Theoretically, an effective preoperative approach can downgrade the tumor stage, facilitate R0 resection, and reduce local relapses and is imperative for patients with potentially resectable gastric cancer (6).

However, it is not clear whether neoadjuvant chemotherapy (NACT) is superior to neoadjuvant chemoradiotherapy (NACRT) in terms of efficacy and safety in potentially resectable gastric carcinoma (7). In 2004, J. A. et al. conducted a multi-institutional trial of NACRT in patients with potentially resectable gastric carcinoma that showed that NACRT caused a substantial pathologic response that resulted in durable survival (8, 9). NACRT followed by surgery and postoperative adjuvant therapy has been clinically recommended for esophageal and gastric junction cancer (10). However, the treatment strategy for non-esophagogastric junction cancer has been controversial, and the application of NACRT for gastric cancer has thus far only been tested in a small number of phase II studies (9). Therefore, in this study, we compared the efficacy and safety of NACRT with those of NACT in resectable gastric cancer through a meta-analysis to provide an evidence-based approach for the treatment of resectable gastric cancer.

## Materials and methods

The Preferred Reporting Items for Systematic Reviews and Meta-Analyses were followed as closely as possible for this systematic review and meta-analysis, and the protocol for this systematic review was registered on the International Platform of Registered Systematic Review and Meta-analysis Protocols (202230164) and is available in full on inplasy.com (https://doi.org/10.37766/inplasy2022.3.0164).

The inclusion criteria of the study were as follows:

i. Randomized controlled trials (RCTs) published worldwide
ii. Patients confirmed by histopathological or cytological examination and assessed by gastroscopy, computed tomography (CT), and magnetic resonance imaging (MRI) to meet the diagnostic criteria for operable gastric cancer
iii. Patients in the experimental group received NACRT, whereas those in the control group received NACT
iv. The objective response rate (ORR), pathologic complete response (pCR), and R0 resection rate were used as primary efficacy outcomes. We evaluated the efficacy of neoadjuvant therapy in the two groups according to the Response Evaluation Criteria in Solid Tumours. Complete response (CR): the disappearance of all target lesions. Partial response (PR): at least a 30% decrease in the sum of diameters of target lesions. Progressive disease (PD): at least a 20% increase in the sum of diameters of target lesions, taking as reference the smallest sum on the study. Stable disease (SD): neither sufficient shrinkage to qualify for PR nor sufficient increase to qualify for PD. ORR: the proportion of patients whose tumors shrank to a certain extent and remained there for a certain time, including CR + PR cases. The secondary indicators were survival rate and incidence of adverse reactions, including nausea and vomiting, myelosuppression, anemia, and digestive tract reactions.

**The exclusion criteria of the study were as follows:**

(i) Review articles, systematic evaluations, animal based experiments, or case reports
(ii) Non-RCTs, observational studies, or retrospective studies
(iii) Repeated articles, studies reporting incomplete or inconsistent outcomes, or having unreasonable trial designs
(iv) Some ongoing clinical trials with no published results
(v) Violation of any of the above inclusion criteria

### Search strategy and study selection

Two investigators (JC and YG) independently searched PubMed, EMbase, Cochrane Library, Web of Science, Chinese National Knowledge Infrastructure, Chinese Biological Medicine Database, Wanfang Database, and VIP Database; we simultaneously searched for related trials in the International Clinical Trial Registry Platform and the Chinese Clinical Registry up to 1 October 2021. We used the following medical subject headings to search for the terms: stomach neoplasms, neoadjuvant chemoradiotherapy, and neoadjuvant chemotherapy. Two investigators filtered the searched articles according to the inclusion and exclusion criteria, and when they had differences, a third researcher determined whether the article would be included.

### Data extraction and quality assessment

Two investigators (JC and YG) independently reviewed the entire articles for all the eligible studies and extracted relevant data, including the author, year of publication, number of patients, age of patients, interventions, radiotherapy dose, and chemotherapy regimen. Two reviewers (MF and YY) evaluated the quality of the selected articles using the Cochrane Collaboration’s tool for RCTs and assessed the items in three categories according to the risk of bias (low, unclear, and high risk of bias), including random sequence generation (selection bias), allocation concealment (selection bias), the blinding of participants and personnel (performance bias), incomplete outcome data (attrition bias), selective reporting (reporting bias), and other biases.

### Statistical analysis

All meta-analyses were performed using Cochrane RevMan version 5.3 and Stata (version 13). The results were reported as pooled odds ratios (ORs) with 95% confidence intervals (95% CIs). We used Cochran’s Q test and I^2^ statistics to evaluate the heterogeneity of all the studies. If the heterogeneity was significant (p < 0.1, I^2^ > 50.0%), the random effects model was adopted; otherwise, the fixed effects model was used. Potential publication bias was assessed using funnel plots, Egger’s test, and Begg’s test. All p-values were two sided, and statistical significance was set at p < 0.05.

## Results

### Characteristics of studies

**Figure 1:**
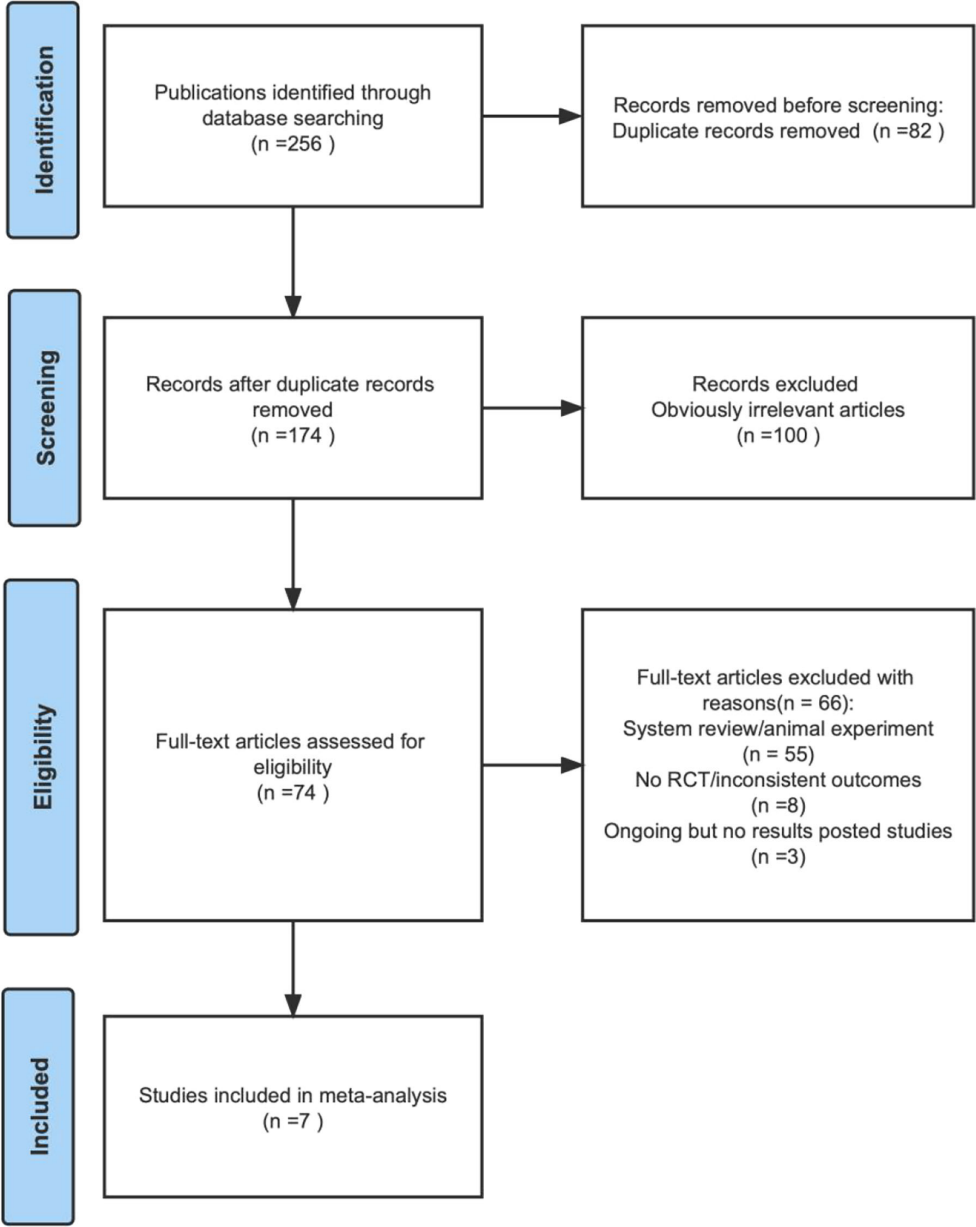
Flow diagram of study selection process for the meta-analysis.

We identified 256 articles for review of the title and abstract (**Figure 1**) and retrieved the full text of potentially eligible articles for a particular assessment after the initial screening. Seven studies were included in the meta-analysis. A total of 601 patients were enrolled, including 302 in the experimental group and 299 in the control group. The particular characteristics of each enrolled article are summarized thoroughly in **Tables 1**–**3**.

**Table 1:** Characteristics of studies enrolled.

Study	Study design	Country	(Experiment/Control) N	Gender (male/female)/N		Age (years)		Tumor location
				Experiment	Control	Experiment	Control	
Cao MF 2019 (11)	RCT (phase II)	China	29 (49)/30(51)	40(68)/19(32)		60.6 ± 7.1		Stomach
Jiang Y 2019 (12)	RCT (phase II)	China	42(50)/42(50)	24(29)/18(21)	25(30)/17(20)	53.14 ± 8.72	53.14 ± 8.72	Stomach (fundus, body, and antrum)
He ZR 2017 (13)	RCT (phase II)	China	25(50)/25(50)	14(28)/11(22)	13(26)/12(24)	46.6 ± 4.5	47.7 ± 4.6	Gastroesophageal junction and the lower and upper third of the stomach
Leong T 2017 (14)	RCT (phase III)	Australia	60(50)/60(50)	45(37)/15(13)	46(38)/14(12)	58 ± 13	56 ± 13	Gastroesophageal junction and the lower and upper third of the stomach
Stahl M 2017 (15, 16)	RCT (phase III)	Germany	60(51)/59(49)	54(45)/6(5)	54(45)/5(4)	Median age 60.6	Median age 56	Gastroesophageal junction
Zhang XT 2016 (17)	RCT (phase II)	China	64 (51)/62(49)	78(62)/48(38)		Median age 55	Median age 57	Stomach (fundus, body, and pylorus)
Wang X 2016 (18)	RCT (phase II)	China	22(51)/21(49)	–	–	–	–	Gastroesophageal junction and the stomach

Data are expressed as n (%).

**Table 2:** Therapeutic regimen of studies enrolled.

Study	RT regimens	Interventions		D-stage resection
		NACRT group	NACT group	
Cao MF 2019 (11)	IMRT (40 Gy/20f/4w)	TC(paclitaxel + carboplatin)+ 40 Gy	TC	D2
Jiang Y 2019 (12)	IMRT (47–50 Gy/24–25f/5–6w)	46.8–50.4 Gy concurrently with capecitabine	Oxaliplatin + capecitabine	–
He ZR 2017 (13)	3D-CRT (45 Gy/25f/5w)	(5-fluorouracil + folinic acid + oxaliplatin)or capecitabine + 45 Gy	(5- fluorouracil + folinic acid + oxaliplatin)or capecitabine	–
Leong T 2017 (14)	3D-CRT or IMRT or VMAT (45 Gy/25f/5w)	(Epirubicin + cisplatin + 5-fluorouracil/capecitabine) + 45 Gy concurrently with 5-fluorouracil/capecitabine	Epirubicin + cisplatin + 5-fluorouracil/capecitabine	D2 recommended, D1 is the minimum approach
Stahl M 2017 (15, 16)	3D-CRT (30 Gy/15f/3w)	5-fluorouracil + folinic acid + cisplatin + 30 Gy with cisplatin and etoposide	5- fluorouracil + folinic acid + cisplatin	D2
Zhang XT 2016 (17)	IMRT (45 Gy/25f/5w)	S-1 + docetaxel + 45 Gy	S-1 + docetaxel	D2
Wang X 2016 (18)	IMRT (45 Gy/22f)	40.04–45.1 Gy concurrently with S-1	SOX (S-1 + oxaliplatin)	–

**Table 3:** The irradiation volumes of studies enrolled.

Study	The irradiation volumes (CTV)
Cao MF 2019 (11)	–
Jiang Y 2019 (12)	–
He ZR 2017 (13)	➢ Tumors of the proximal third of the stomach or cardiac esophagogastric junction: primary tumor, 3–5 cm of the lower esophagus, the left hemidiaphragm, and the adjacent pancreatic body, with high-risk lymph node areas including the adjacent peri-e,sophageal, perigastric, suprapancreatic, celiac trunk, splenic artery and splenic hilar lymph node areas. ➢ Tumors of the middle third of the stomach or the body of the stomach: primary tumor and the pancreatic body, with the lymph node area including the adjacent perigastric, suprapancreatic, truncal and splenic hilar, hepatic, and duodenal lymph node areas. ➢ Tumors of the distal third of the stomach: if the gastroduodenal junction is involved: primary tumor, the head of the pancreas, the first and second segments of the duodenum, with the lymph node area including the perigastric, suprapancreatic, celiac trunk, hilar, and pancreaticoduodenal lymph nodes.
T.Leong 2017 (14)	➢ The entire stomach, any perigastric tumor extension, and regional lymph nodes.
M.Stahl 2017 (15, 16)	➢ The pretherapeutic extension of the primary tumor with a transversal margin of 2 cm and a both- sides longitudinal margin along the mucosa of the gastro-oesophageal junction (GEJ) of 5 cm in Siewert type 1 tumors. ➢ Suspicious lymph nodes with a 1-cm margin and the regional lymph nodes with a margin of 1.5 cm around the cardia, along the left gastric artery and the minor curvature to the incisura angularis, the celiac artery, the proximal part of the commune hepatic artery, and along the first 2 cm of the splenic artery.
Zhang XT 2016 (17)	–
X.Wang 2016 (18)	–

### Quality assessment

**Figure 2:**
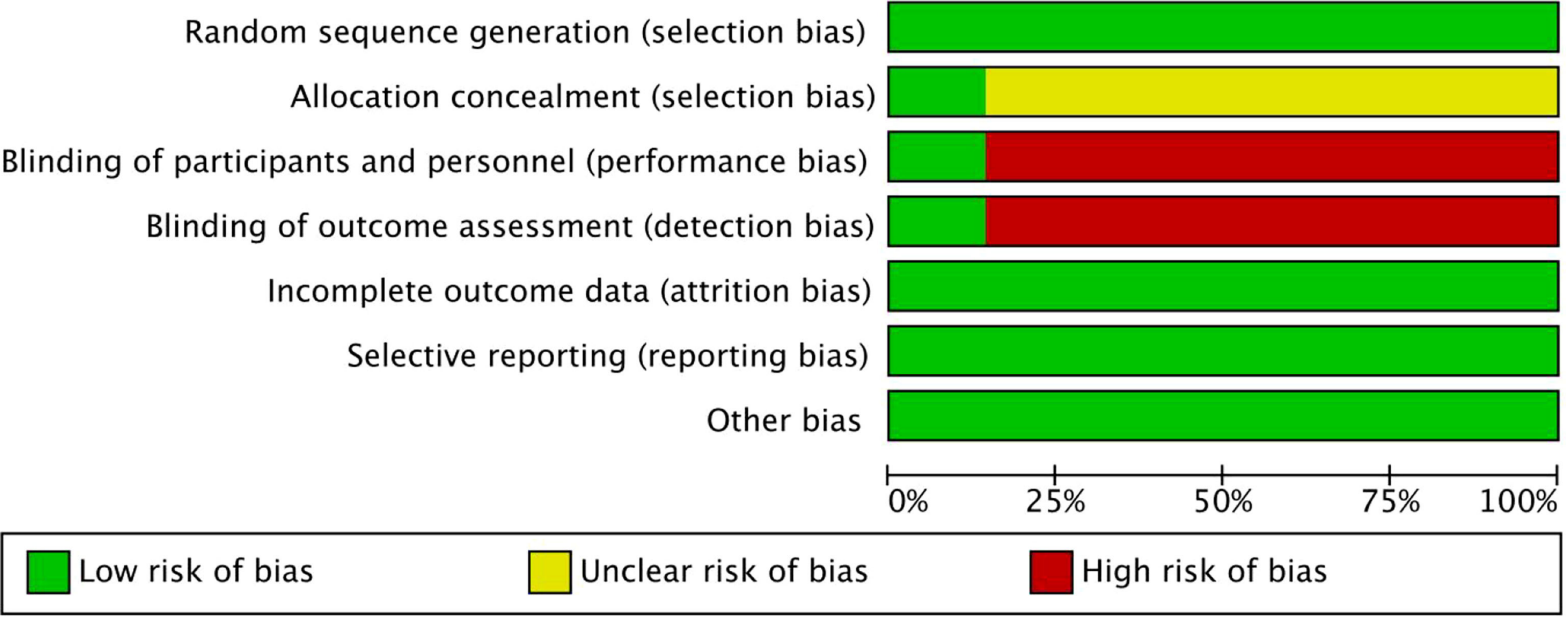
Risk of bias graph for seven randomized controlled trials (RCTs).

**Figure 3:**
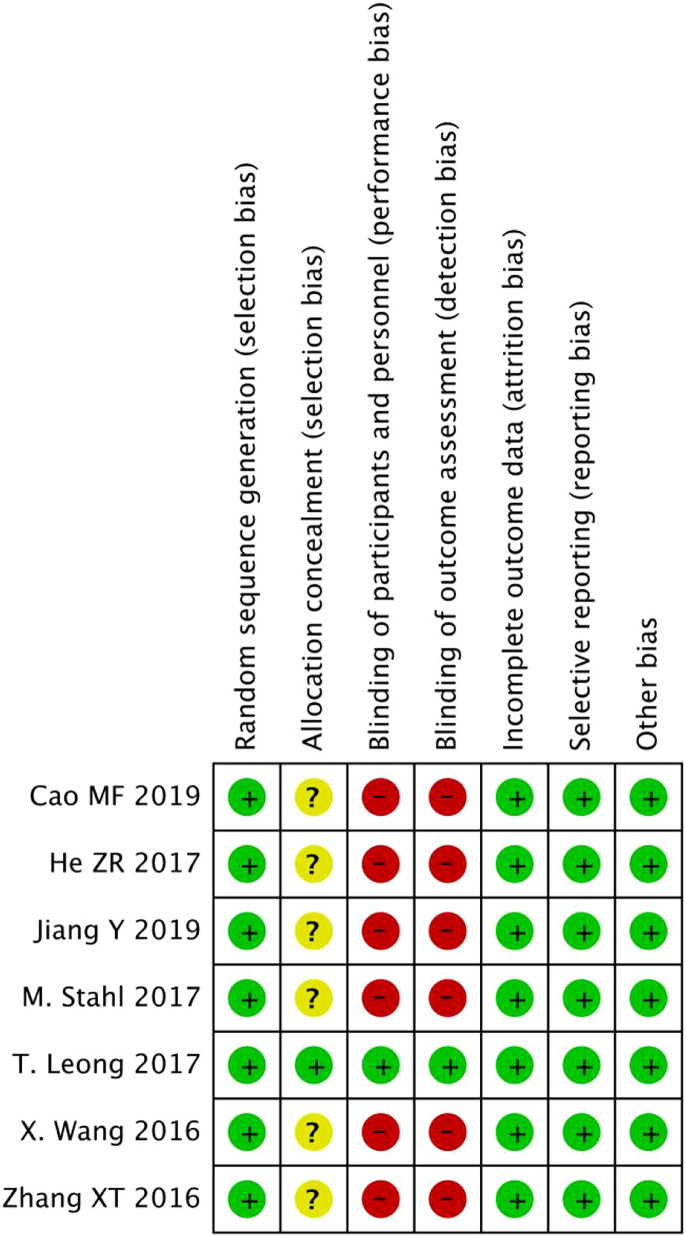
Risk of bias summary for seven RCTs.

We evaluated the quality of all meta-analyses using the Cochrane Collaboration’s tool for assessing the risk of bias, as shown in **Figures 2**, **3**. Through our assessment, we concluded that all the included articles were randomized controlled trials, of which one article followed allocation concealment and other articles included trials carried out using the method of informed consent. There were no errors in that all the eligible studies adopted random numbers to decide the final treatment and all had completed data, no selective reports, or other deviations.

### Efficiency

#### Complete response

**Figure 4:**
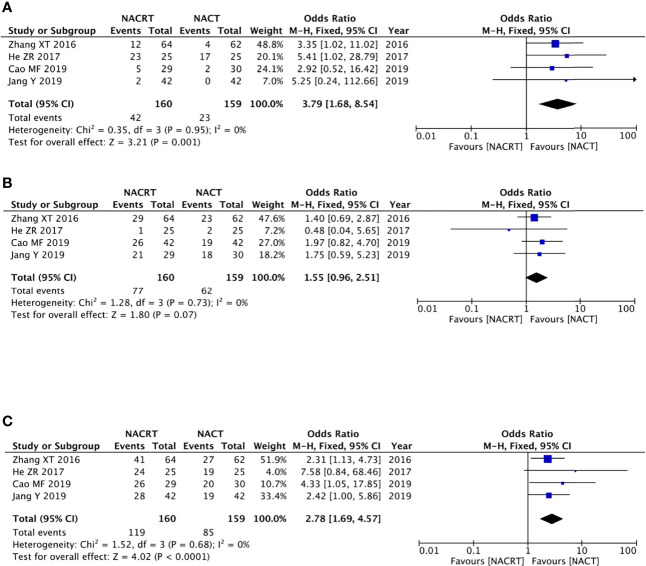
Forest plot for the complete response (CR) **(A)**, partial response (PR) **(B)**, and objective response rate (ORR) **(C)** of the neoadjuvantchemoradiotherapy (NACRT) group and neoadjuvant chemotherapy (NACT) group.

Four of the included articles reported the CR. Because there was no heterogeneity between the studies (p = 0.95, I^2^ = 0%), we adopted the fixed effects model for meta-analysis, which showed that the CR rate in the NACRT group was higher than that in the NACT group (OR = 3.79, 95% CI: 1.68–8.54, p = 0.001) and that the results were statistically significant (**Figure 4A**).

#### Partial response

Four of the included articles reported the PR. Because there was no heterogeneity between the studies (p = 0.73, I^2^ = 0%), we adopted the fixed effects model for meta-analysis, which showed that the results were not statistically significant (OR = 1.55, 95% CI: 0.96–2.51, p = 0.07) (**Figure 4B**).

#### Objective response rate

There were four studies that reported the ORR. There was no heterogeneity between the studies (p = 0.68, I^2^ = 0%); we therefore adopted the fixed effects model for meta-analysis, which showed that the ORR rate in the NACRT group was higher than that in the NACT group (OR = 2.78, 95% CI: 1.69–4.57, p < 0.0001) and that the results were statistically significant (**Figure 4C**).

#### Pathologic complete response rate

**Figure 5:**
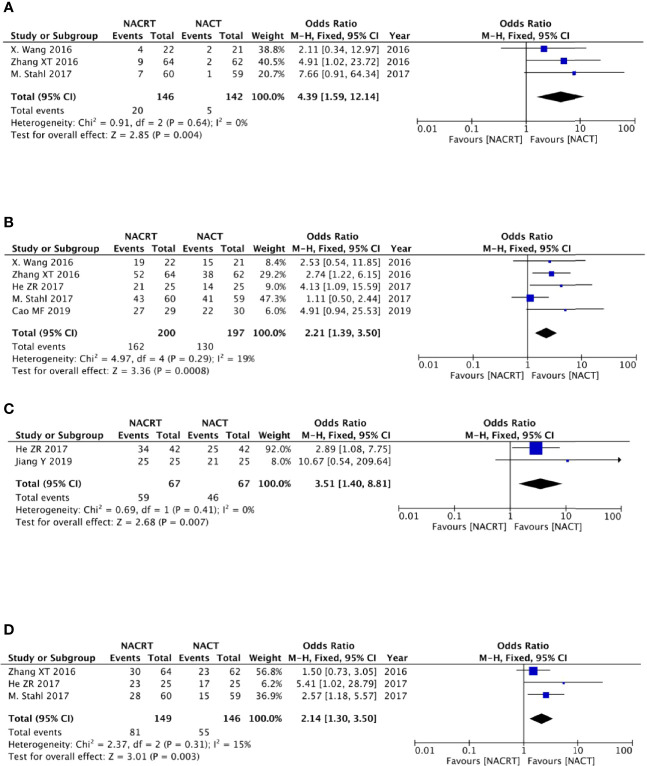
Forest plot for the pathologic complete response (pCR) rate **(A)**, R0 resection rate **(B)**, and 1- and 3-year survival rates **(C, D)**.

There were three studies among the included articles that reported the pCR. We adopted the fixed effects model for meta-analysis because there was no heterogeneity between the studies (p = 0.64, I^2^ = 0%), which showed that the pCR rate in the NACRT group was higher than that in the CRT group (OR = 4.39, 95% CI: 1.59–12.14, p = 0.004) and that the results were statistically significant (**Figure 5A**).

#### R0 resection rate

Of the included articles, five studies reported R0 resection rates. No heterogeneity was observed between the studies (p = 0.29, I^2^ = 19%); we therefore adopted the fixed effects model for meta-analysis, which showed that the R0 resection rate in the NACRT group was higher than that in the NACT group (OR = 2.21, 95% CI: 1.39–3.50, p = 0.0008) and that the results were statistically significant (**Figure 5B**).

#### 1-year and 3-year survival rates

Two studies reported the 1-year survival rate, and three studies reported the 3-year survival rate. Due to the lack of heterogeneity between the studies (p = 0.41, I^2^ = 0% and p = 0.31, I^2^ = 15%), we adopted the fixed effects model for meta-analysis, which showed that the 1-year survival rate in the NACRT group was higher than that in the NACT group (OR = 3.51, 95% CI: 1.40–8.81, p = 0.007), and the 3-year survival rate in the NACRT group was also higher than that in the NACT group (OR = 2.14, 95% CI: 1.30–3.50, p = 0.003). The results were all statistically significant (**Figures 5C**, **D**).

### Postoperative complications

**Figure 6:**
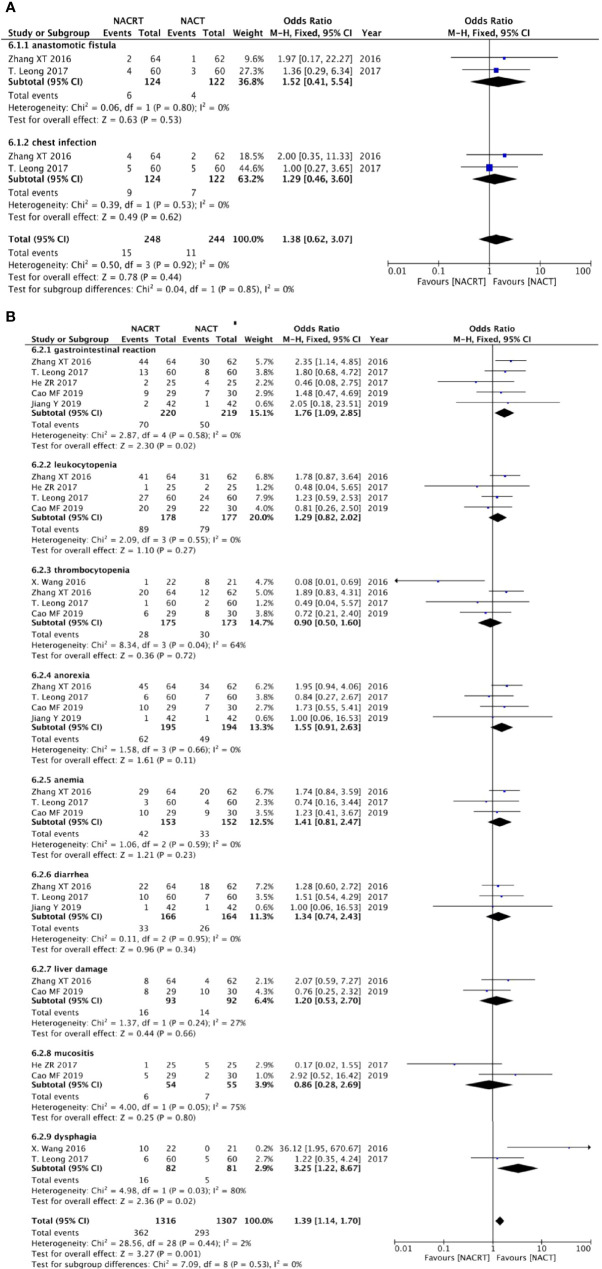
Forest plot for postoperative complications **(A)** and adverse effects after neoadjuvant therapy **(B)**.

Two of the included articles reported anastomotic leak, and two studies reported abdominal infection. Because no heterogeneity was found between the studies (p = 0.80, I^2^ = 0% and p = 0.53, I^2^ = 0%), we adopted the fixed effects model for meta-analysis, which showed that there was no difference in the incidence of anastomotic leak and abdominal infection between the two groups (**Figure 6A**).

### Adverse effects after neoadjuvant therapy

There were five studies that reported gastrointestinal reaction, four studies reported leukocytopenia, four studies indicated thrombocytopenia, four studies reported anorexia, three reported anemia, three indicated diarrhea, two studies mentioned liver damage, two studies reported mucositis, and two studies indicated dysphagia. The results showed that there was no statistical significance in the incidence of adverse reactions, except gastrointestinal reactions that were higher in the NACRT group than in the NACT group (OR = 1.76, 95% CI: 1.09–2.85, p = 0.02), and this result was statistically significant (**Figure 6B**).

### Sensitivity and publication bias evaluation

**Figure 7:**
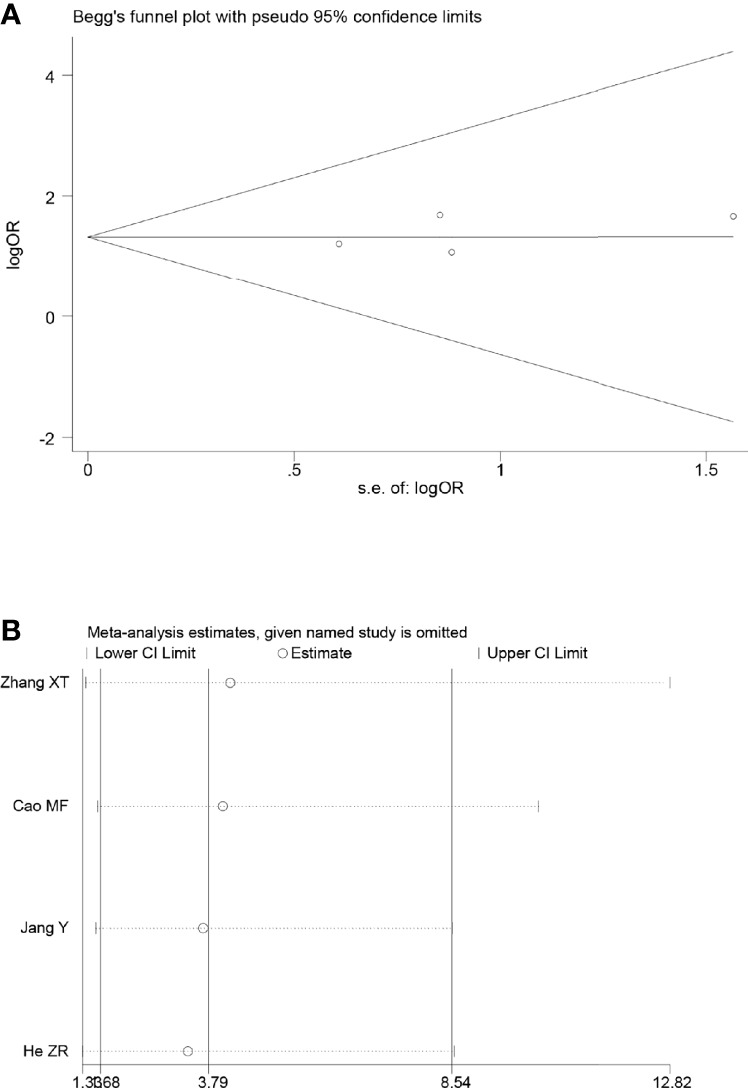
Begg’s funnel plot **(A)** and sensitivity analysis **(B)** of all the included studies for the analysis of CR.

**Figure 8:**
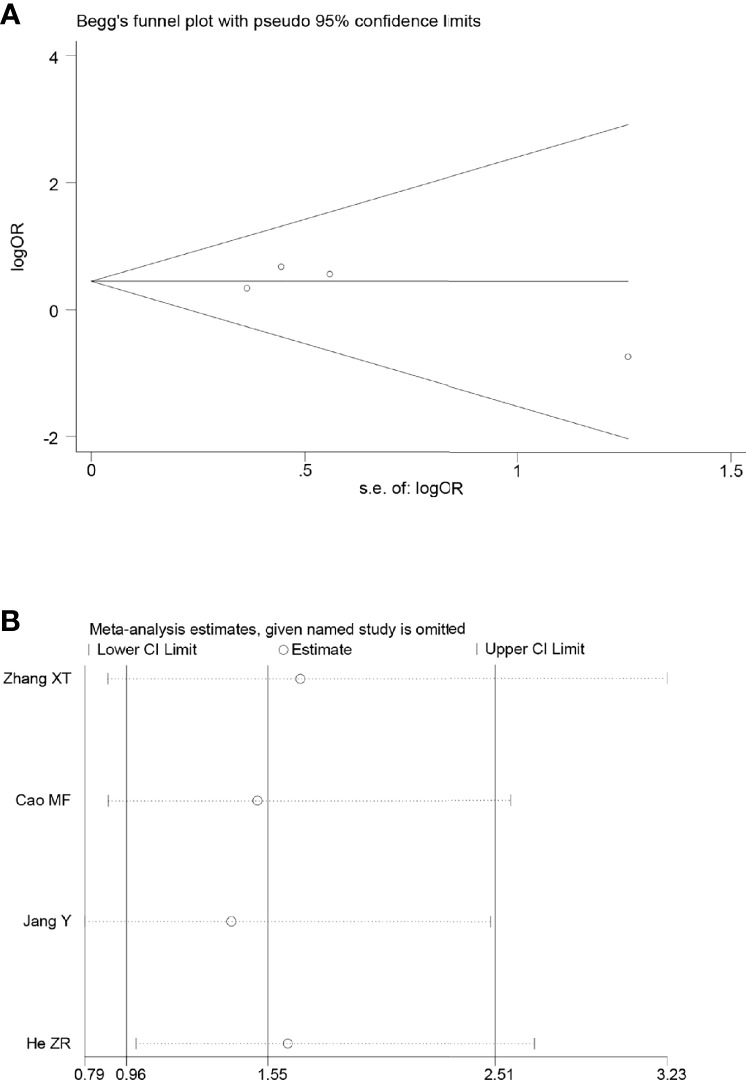
Begg’s funnel plot **(A)** and sensitivity analysis **(B)** of all the included studies for the analysis of the R0 resection rate.

**Figure 9:**
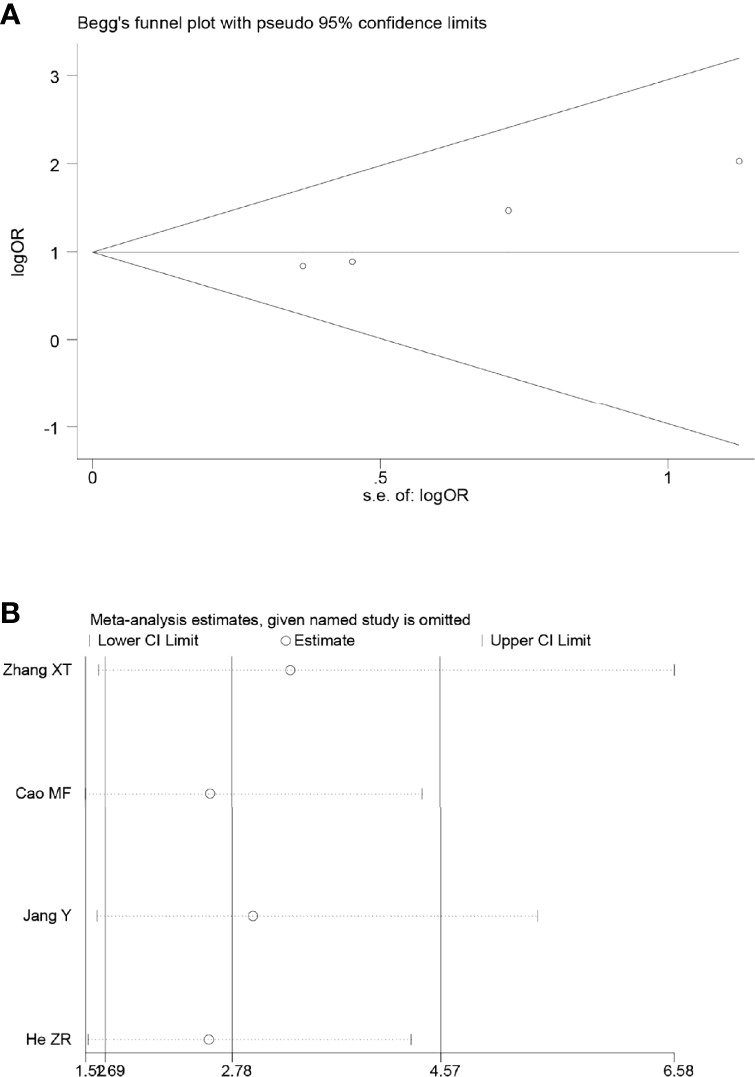
Begg’s funnel plot **(A)** and sensitivity analysis **(B)** of all the included studies for the analysis of PR. .

**Figure 10:**
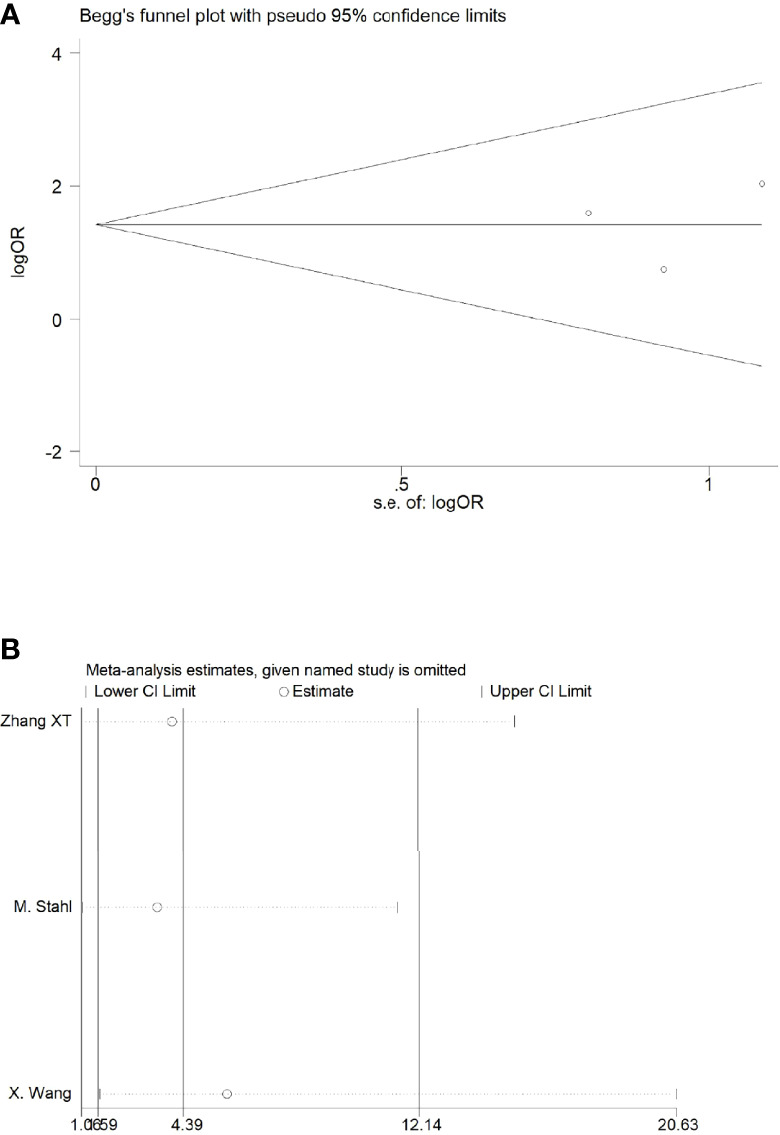
Begg’s funnel plot **(A)** and sensitivity analysis **(B)** of all the included studies for the analysis of ORR. .

**Figure 11:**
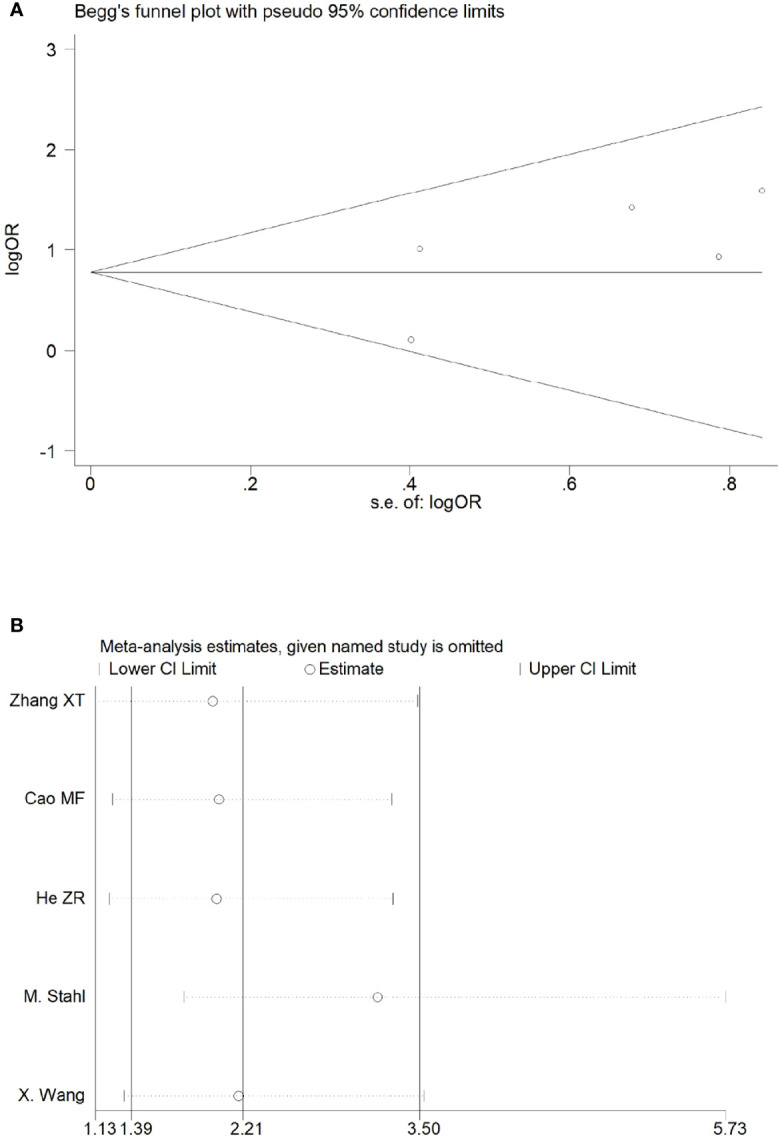
Begg’s funnel plot **(A)** and sensitivity analysis **(B)** of all the included studies for the analysis of the pCR rate.

Sensitivity analyses were performed by excluding one study at a time, to assess the influence of each study on the overall results. The results showed that the deletion of any one study had no significant effect on the results (**Figures 7B**–**11**B), indicating that the results of this meta-analysis are relatively stable. The publication bias analysis of the seven included articles showed that there was no obvious publication bias in the CR, PR, ORR, pCR rate, and R0 resection rate. Begg’s funnel plot indicated no significant publication bias (**Figures 7A**–**11**A).

## Discussion

Our study supports the efficacy and safety of NACRT compared to NACT for resectable gastric cancer. Neoadjuvant therapy is effective in reducing the volume of the primary tumor, tumor stage, and lymph node involvement to narrow the range of surgical resection, improve the R0 resection rate, and prolong the survival cycle (19, 20). In addition, neoadjuvant therapy can reduce or eliminate the risk of residual tumor cells and distant metastasis, which are considered to be closely associated with postoperative recurrence and metastasis. Some studies have also shown that pathological reactions after neoadjuvant therapy are closely associated with a reduction in the recurrence rate and overall survival (21–27). Neoadjuvant chemoradiotherapy + surgery + postoperative adjuvant chemotherapy has become the standard treatment for resectable esophagogastric junction cancer (10). However, the choice of preoperative neoadjuvant therapy for non-esophagogastric junction cancer remains controversial (28, 29). Whether neoadjuvant chemotherapy should be combined with radiotherapy requires more clinical studies to prove its efficacy and safety.

This systematic review included seven RCTs involving 601 patients. The results of our study showed that the NACRT group had an increased number of patients with CR, ORR, and pCR; improved R0 resection rate; and 1-year and 3-year survival rates. In our meta-analysis, the average ORR rate of the NACRT group in the four enrolled articles was 79.1%, compared to 57.9% in the NACT group, and the highest ORR rate was 96% in the study by He ZR (13). Of the seven studies, five reported R0 resection rates; the average R0 resection rate was 83.28% in the NACRT group and 66.31% in the NACT group. In terms of 1-year and 3-year survival rates, the NACRT group had higher survival rates than the NACT group, and the results were statistically significant. Two of the included studies reported the median survival time; the NACRT group had a significantly longer median survival time [27.5 m *vs*. 22.5 m in the study by Zhang XT (17), and 30.8 m *vs*. 21.1 m in the study by Stahl M (15)] These results provide sufficient evidence for the efficacy of NACRT in resectable gastric cancer. Moreover, there was no difference in the incidence of adverse effects (except for the occurrence rate of gastrointestinal reactions) and postoperative complications between the two groups after neoadjuvant therapy. In conclusion, it stands to reason that the patients of resectable gastric cancer benefit from NACRT.

Some challenges remain before NACRT can become a standard treatment strategy. First, the adjuvant and neoadjuvant therapies have always been complementary. Results from the CRITIC study of chemotherapy versus chemoradiotherapy after surgery and preoperative chemotherapy for resectable gastric cancer showed that postoperative chemoradiotherapy did not improve overall survival (30). However, in the current analysis, only patients who started their allocated postoperative treatment were included, and the per-protocol (PP) analysis of patients who started the allocated postoperative treatment showed that the chemotherapy group had a significantly better 5-year overall survival than the chemoradiotherapy group (31). This study was based on adjuvant therapy administered after neoadjuvant chemotherapy. If neoadjuvant chemoradiotherapy is widely used, the choice of postoperative adjuvant therapy should be explored. Second, there are likely biological differences between Eastern and Western countries. Most of our studies were from China, and whether NACRT works for Westerners remains unknown (32). Furthermore, as mentioned above, NACRT is proven to be effective for resectable esophagogastric junction cancers, and the current debate is only about non-esophagogastric junction cancers. Some of our enrolled studies did not clearly define non-esophagogastric junction cancer as the inclusion criteria that might have caused some discrepancy in our research.

### Limitations

This meta-analysis has certain limitations. First, although the included studies were all RCTs, the sample size of some studies was small. Second, the interventions of the enrolled studies, the chemotherapy regimen, or the recommended dose of radiotherapy were inconsistent, which may have caused some degree of bias. The outcome indicators mentioned in this article are not identical. Jiang Y regarded the ORR as the primary efficacy outcome and not the R0 resection rate (12). Leong T [the Trial Of Preoperative therapy for Gastric and Esophagogastric junction AdenocaRcinoma (TOPGEAR)] only reported the interim results regarding adverse effects after neoadjuvant therapy and postoperative complications, whereas we expected the final results of this randomized, phase III trial (14). Several ongoing studies have not published their results (such as the PREACT trial), and we believe that their final results will help our research (33).

## Conclusion

In conclusion, our meta-analysis demonstrated the efficacy and safety of NACRT for resectable gastric cancer, providing clinical support for its wide application. However, since some clinical trials have not yet reached their end points, the long-term outcomes and toxicity must be examined to confirm this conclusion.

## Data availability statement

The original contributions presented in the study are included in the article/**Supplementary Material**. Further inquiries can be directed to the corresponding author.

## Author contributions

JC, YX, and LZ contributed to the conception and design of the study. JC and YY organized the databases and provided methodological support. YG, MF, and YZ performed the statistical analysis. All authors contributed to the article and approved the submitted version.

## Funding

This work was supported by a key program from the National Natural Science Foundation of China under Grant 81972845; Introduction of Specialist Team in Clinical Medicine of Xuzhou under Grant 2019TD003; Postgraduate Research & Practice Innovation Program of Jiangsu Province (SJCX22_1273).

## Conflict of interest

The authors declare that the research was conducted in the absence of any commercial or financial relationships that could be construed as a potential conflict of interest.

## Publisher’s note

All claims expressed in this article are solely those of the authors and do not necessarily represent those of their affiliated organizations, or those of the publisher, the editors and the reviewers. Any product that may be evaluated in this article, or claim that may be made by its manufacturer, is not guaranteed or endorsed by the publisher.
