# Supplementary material for: Neoadjuvant chemoradiotherapy for resectable gastric cancer: A meta-analysis
Source: Front Oncol. 2022 Aug 5;12:927119. doi: 10.3389/fonc.2022.927119 (PMC9388908; doi:10.3389/fonc.2022.927119)
Supplement: Supplementary file 1 [file DataSheet_1.docx]

Supplementary Material

# Supplementary materials caption

**TABLE S1** | Summary of the meta-analysis

**Figure S1** | Publication bias of the recruited literatures. Funnel plot of the analysis of CR(A), PR(B), ORR(C), pCR rate(D), R0 resection rate(E).

**Figure S2** | Egger’s plot of the analysis of publication bias of CR(A), PR(B), ORR(C), pCR rate(D), R0 resection rate(E).

**Figure S3** | Begg’s and Egger’s tests for publication bias of CR(A), PR(B), ORR(C), pCR rate(D), R0 resection rate(E).

| Outcomes and toxicity | Included study | Number of  Experience | Number of  Control | Heterogeneity | | Meta-analysis model | Result of meta-analysis | |
| --- | --- | --- | --- | --- | --- | --- | --- | --- |
|  |  |  |  | P | I^2^ |  | OR(95%CI) | P |
| CR | 4 | 160 | 159 | 0.95 | 0% | fixed-effects | 3.79(1.68-8.54) | 0.001 |
| PR | 4 | 160 | 159 | 0.73 | 0% | fixed-effects | 1.55(0.96-2.51) | 0.07 |
| ORR | 4 | 160 | 159 | 0.68 | 0% | fixed-effects | 2.78(1.69-4.57) | <0.0001 |
| pCR rate | 3 | 146 | 142 | 0.64 | 0% | fixed-effects | 4.39(1.59-12.14) | 0.004 |
| R0 resection rate | 5 | 200 | 197 | 0.29 | 19% | fixed-effects | 2.21(1.39-3.50) | 0.0008 |
| 1-year survival rate | 2 | 67 | 67 | 0.41 | 0% | fixed-effects | 3.51(1.40-8.81) | 0.007 |
| 3-year survival rate | 3 | 149 | 146 | 0.31 | 15% | fixed-effects | 2.14(1.30-3.50) | 0.003 |
| **postoperative complications** |  |  |  |  |  |  |  |  |
| Anastomotic fistula | 2 | 124 | 122 | 0.8 | 0% | fixed-effects | 1.52(0.41-5.54) | 0.53 |
| Chest infection | 2 | 124 | 122 | 0.53 | 0% | fixed-effects | 1.29(0.46-3.60) | 0.62 |
| **adverse effects after neoadjuvant therapy** |  |  |  |  |  |  |  |  |
| gastrointestinal reaction | 5 | 220 | 219 | 0.58 | 0% | fixed-effects | 1.76(1.09-2.85) | 0.02 |
| leukocytopenia | 4 | 178 | 177 | 0.55 | 0% | fixed-effects | 1.29(0.82-2.02) | 0.27 |
| anorexia | 4 | 195 | 194 | 0.66 | 0% | fixed-effects | 1.55(0.91-2.63) | 0.11 |
| anemia | 3 | 153 | 152 | 0.59 | 0% | fixed-effects | 1.41(0.81-2.47) | 0.23 |
| dirarrhea | 3 | 166 | 164 | 0.95 | 0% | fixed-effects | 1.34(0.74-2.43) | 0.34 |
| liver damage | 2 | 16 | 14 | 0.24 | 27% | fixed-effects | 1.20（0.53-2.70） | 0.66 |

**TABLE S1**

## Figure S1


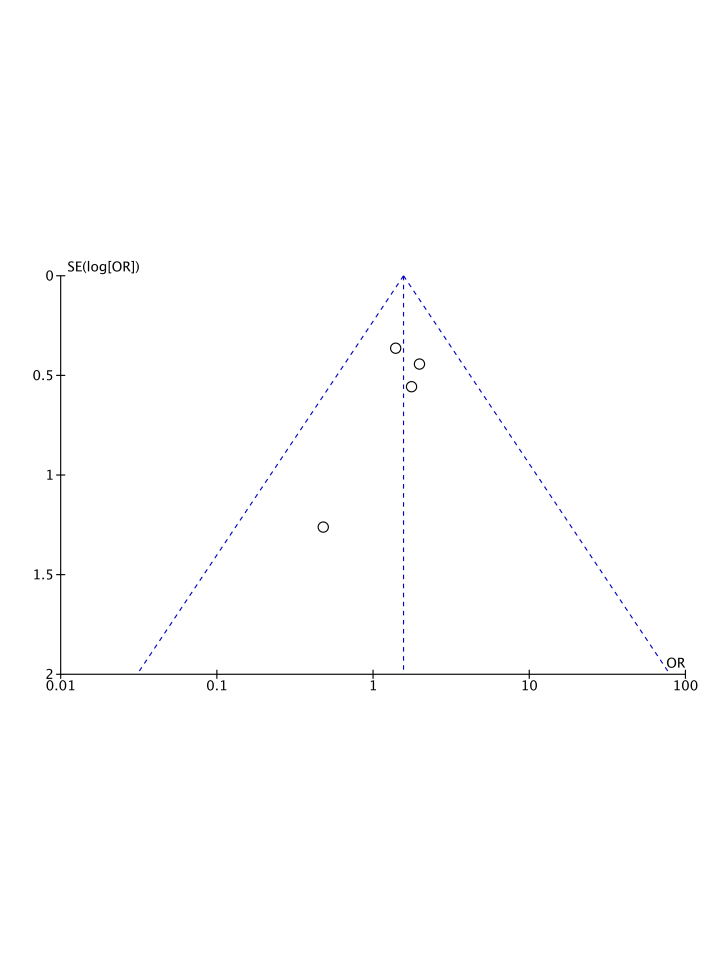

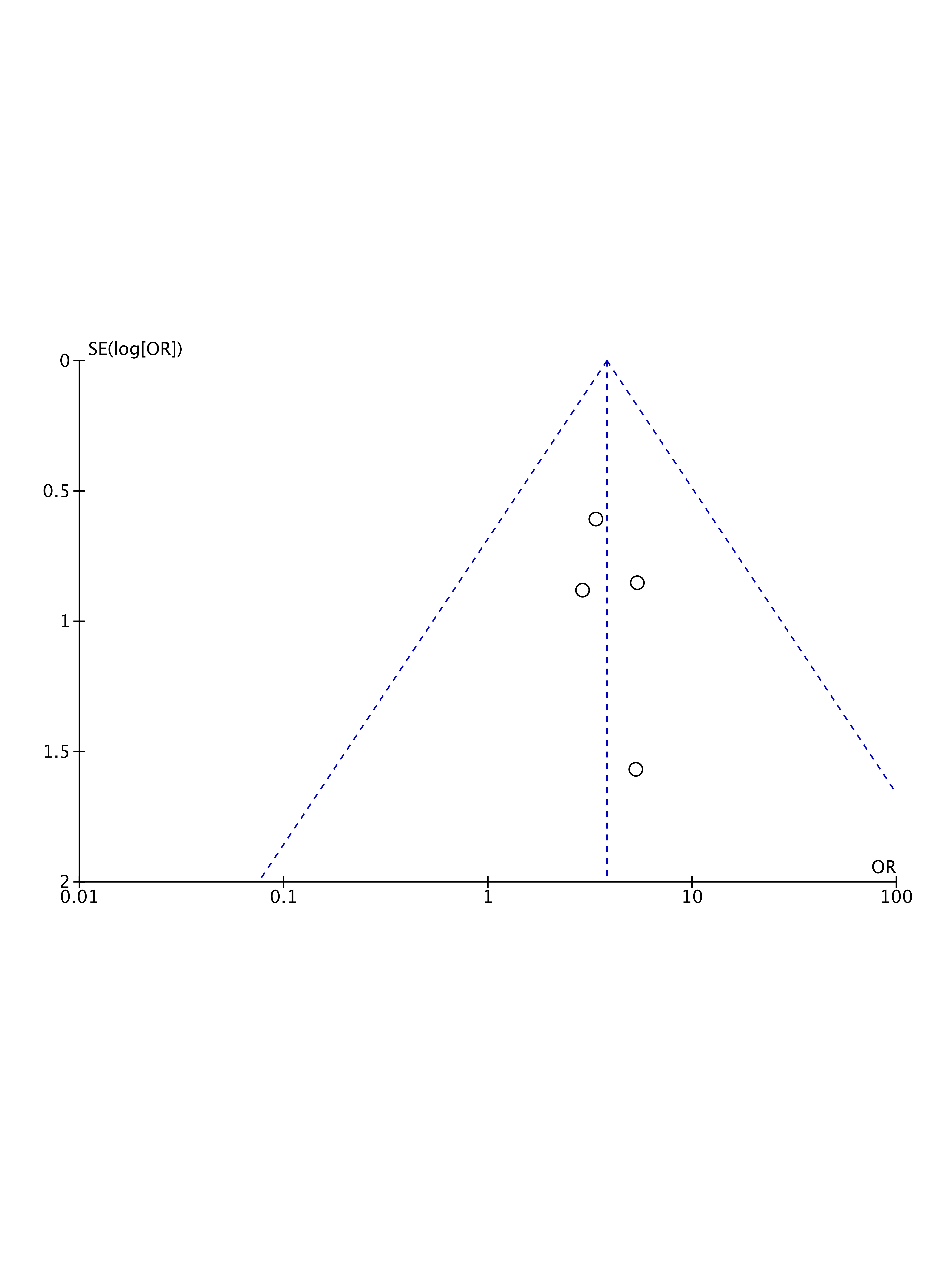
A B

C D


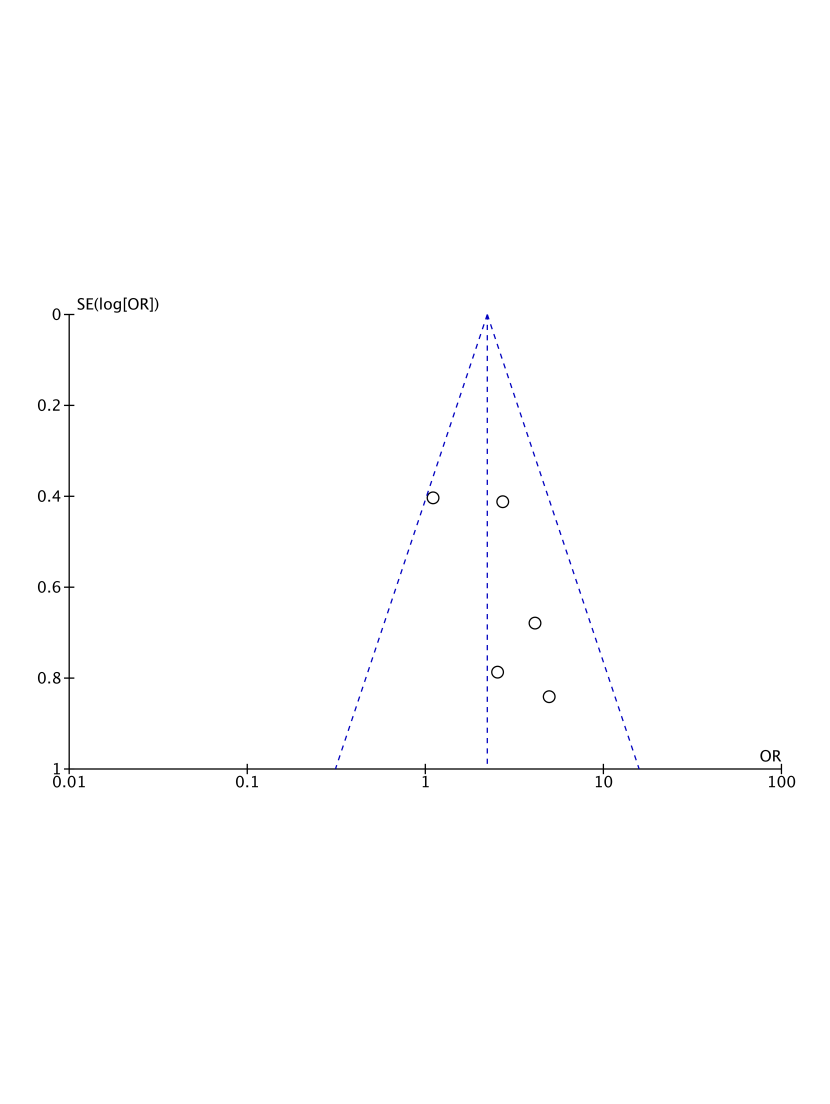

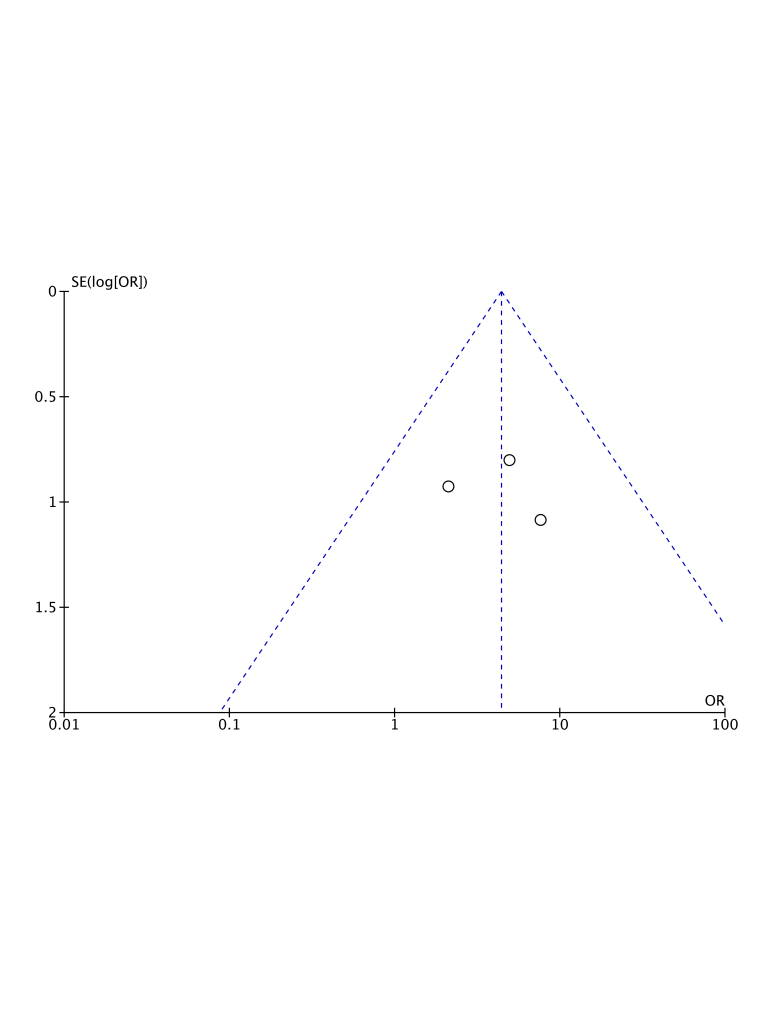

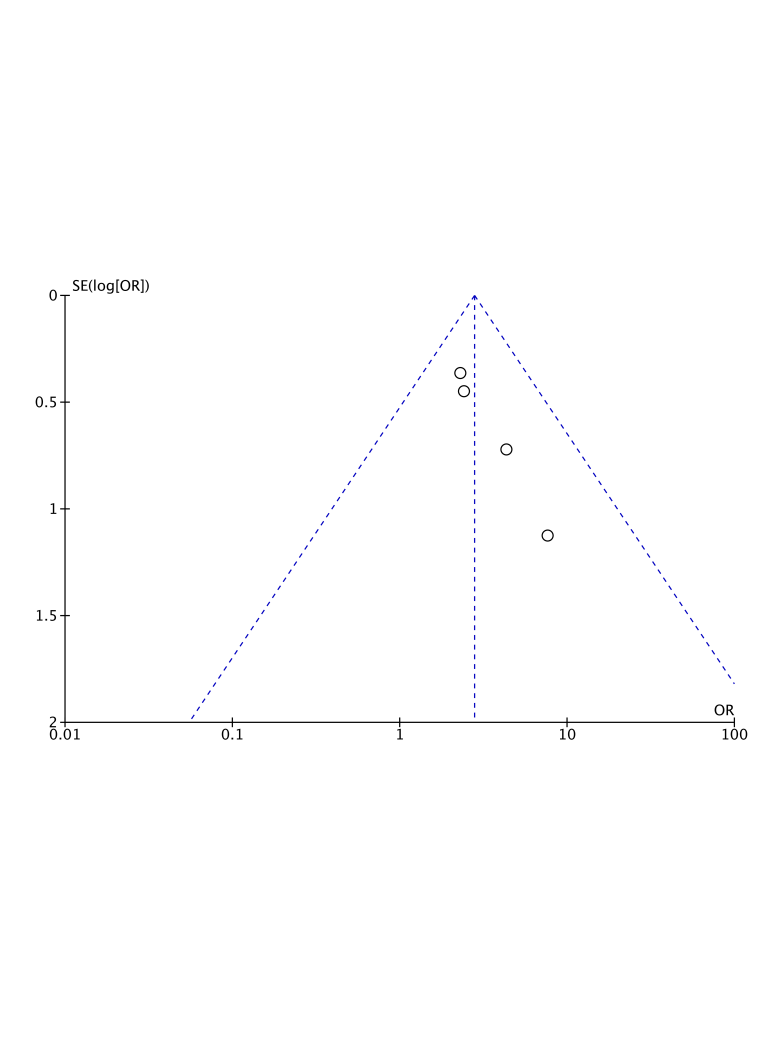
E

## Figure S2

A B

****C D


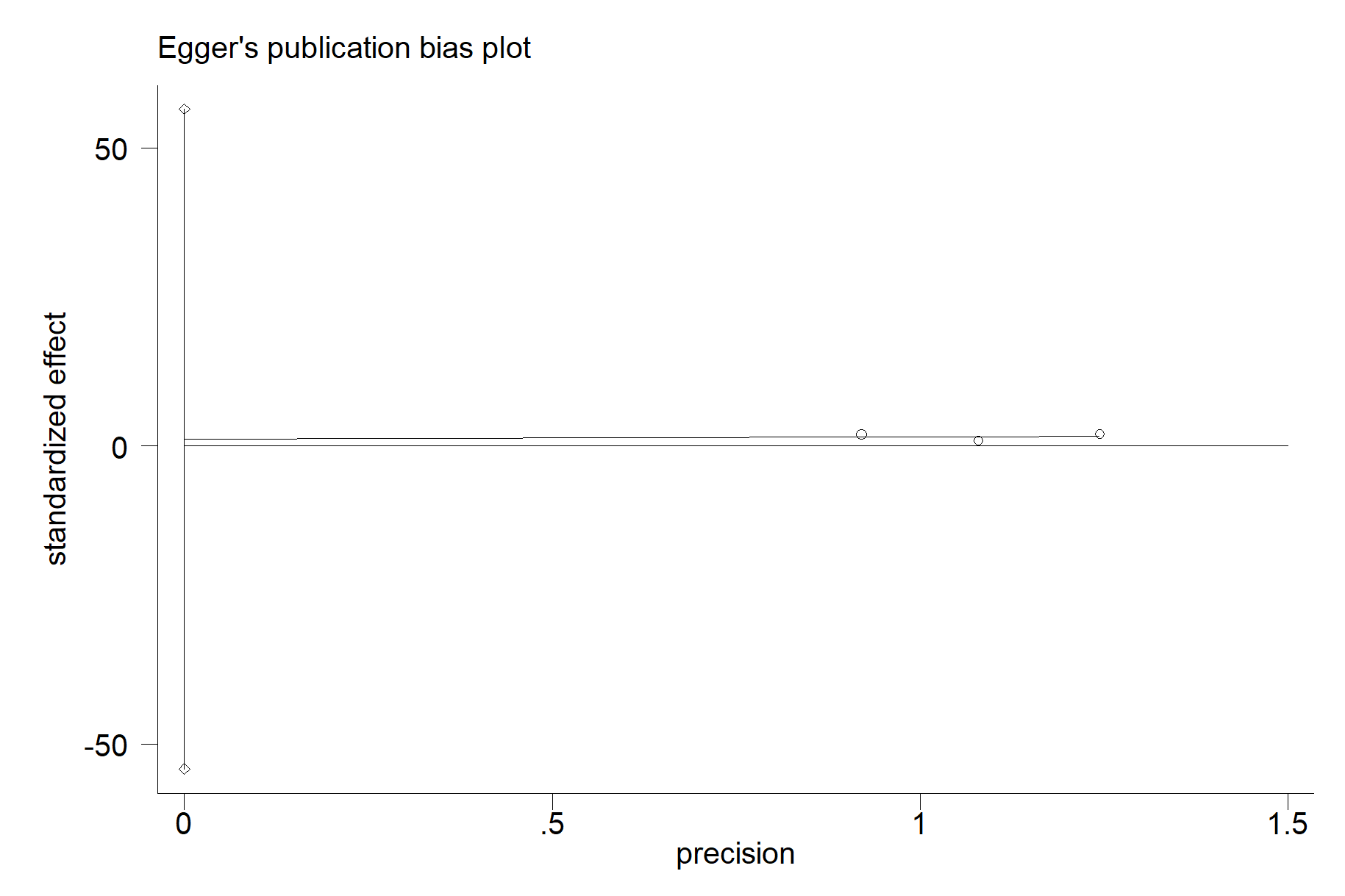
E


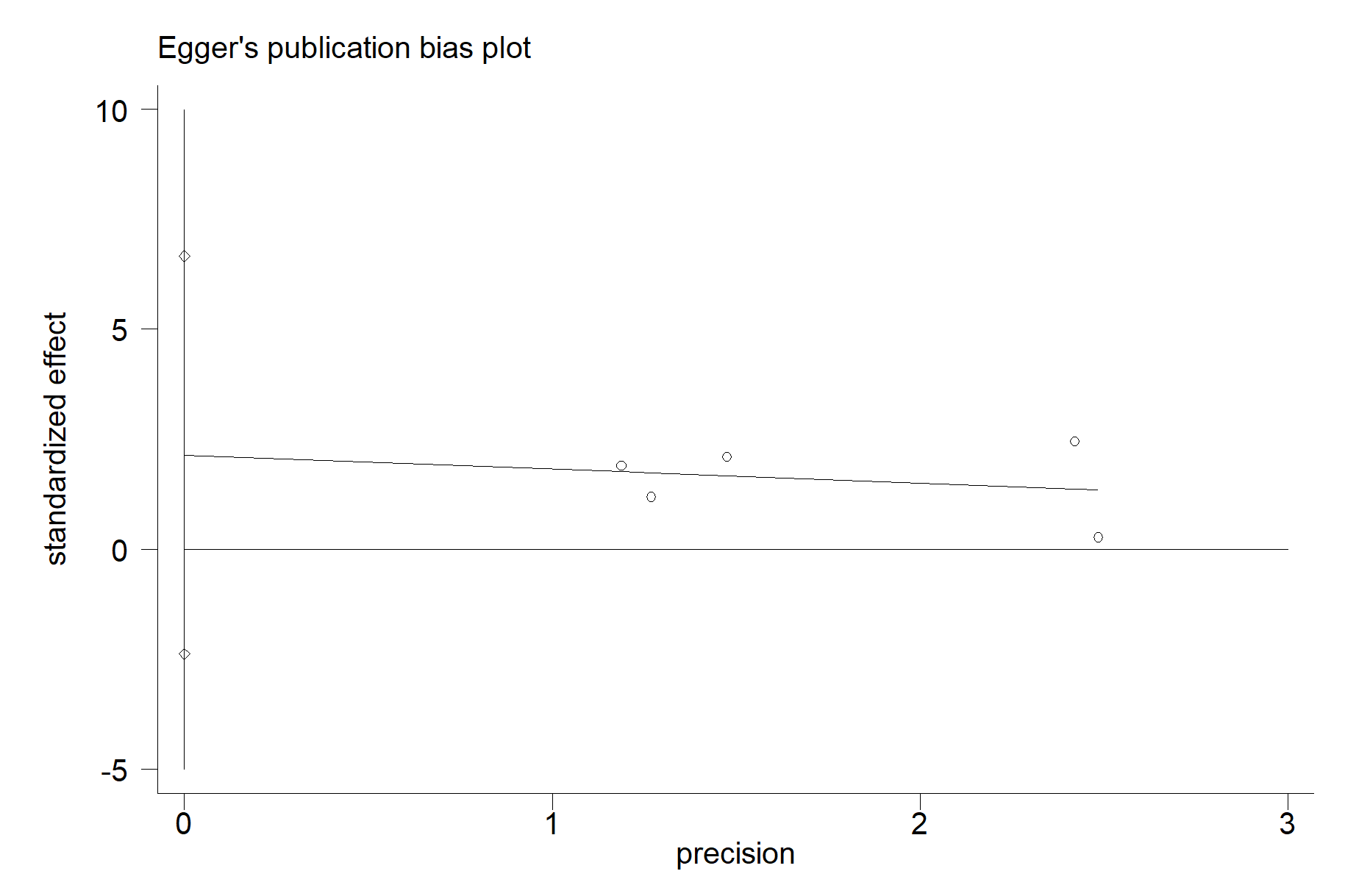


## Figure S3


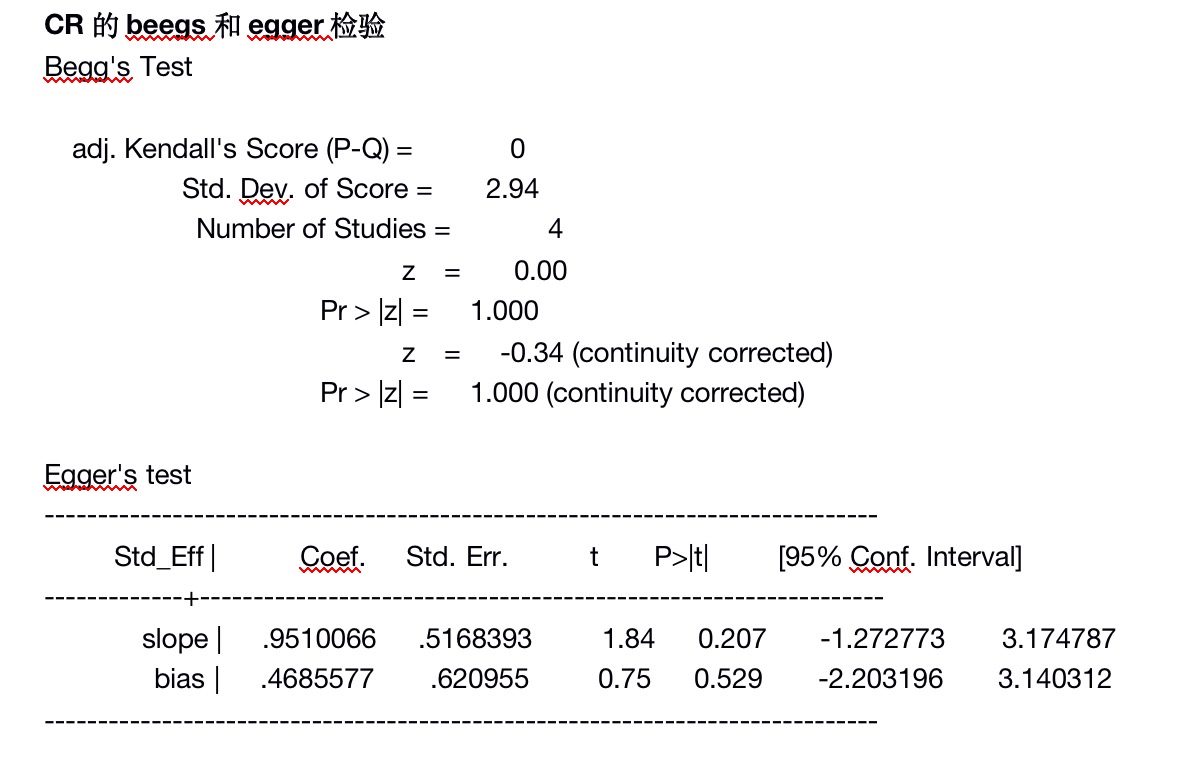

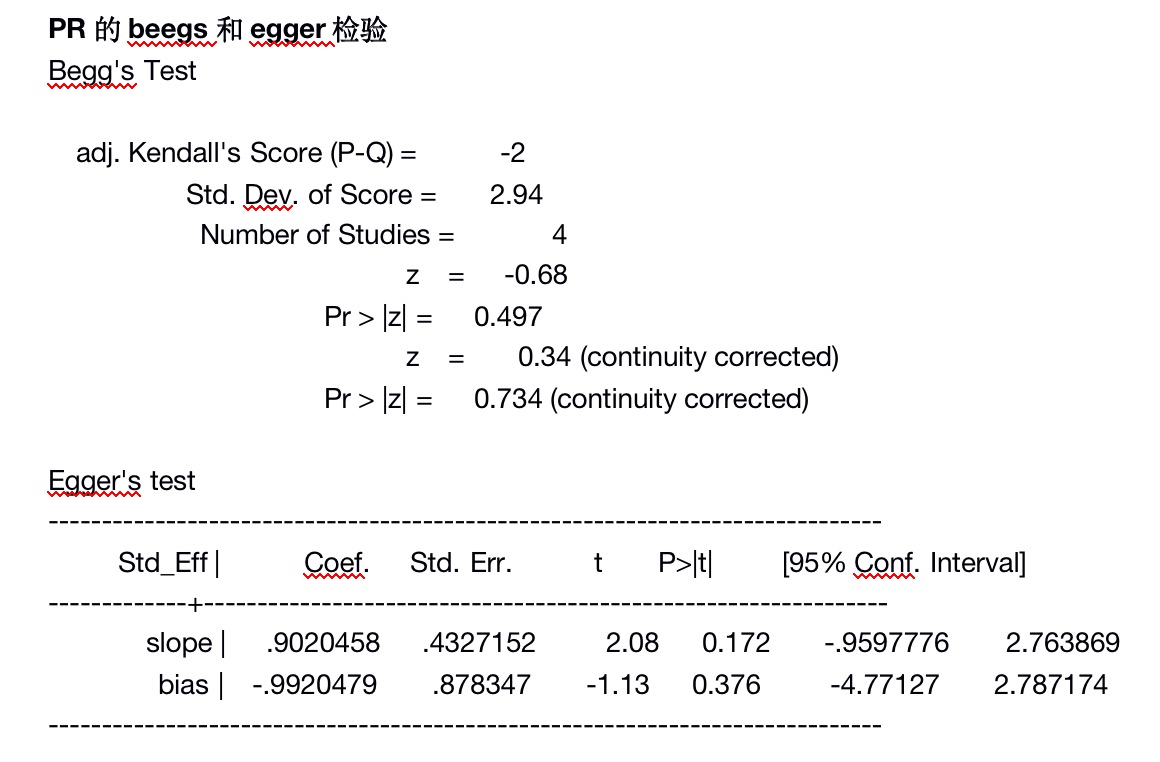
A B

C D


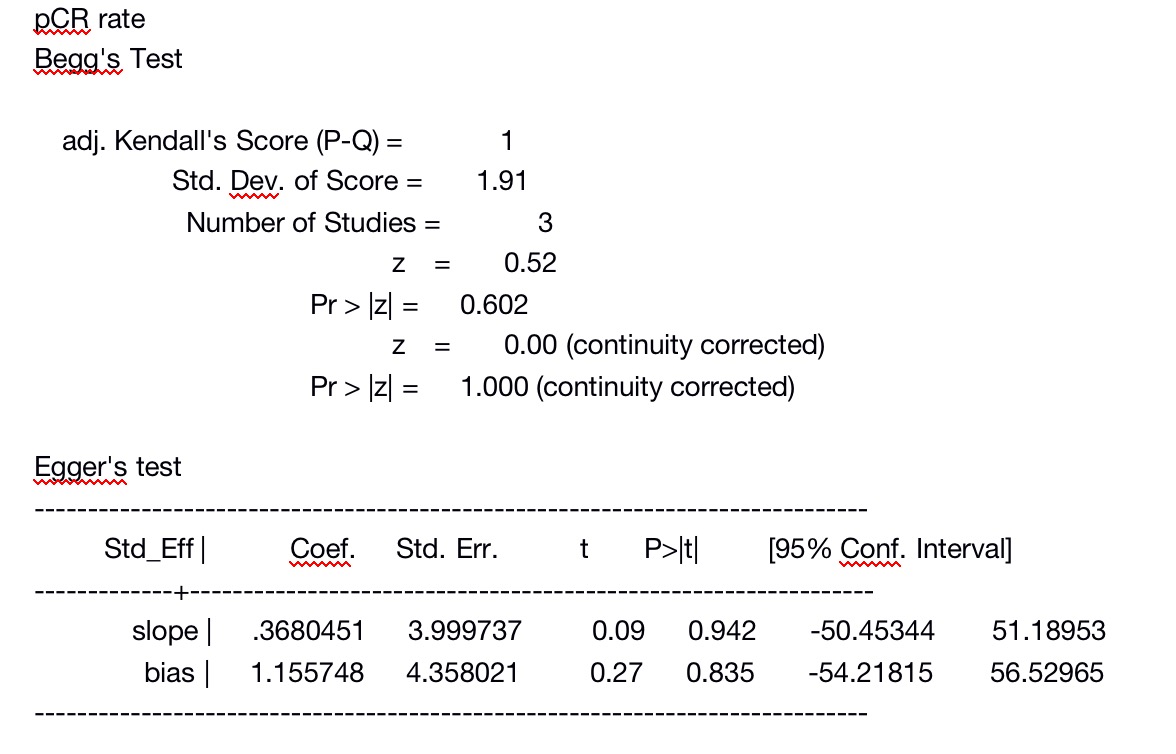

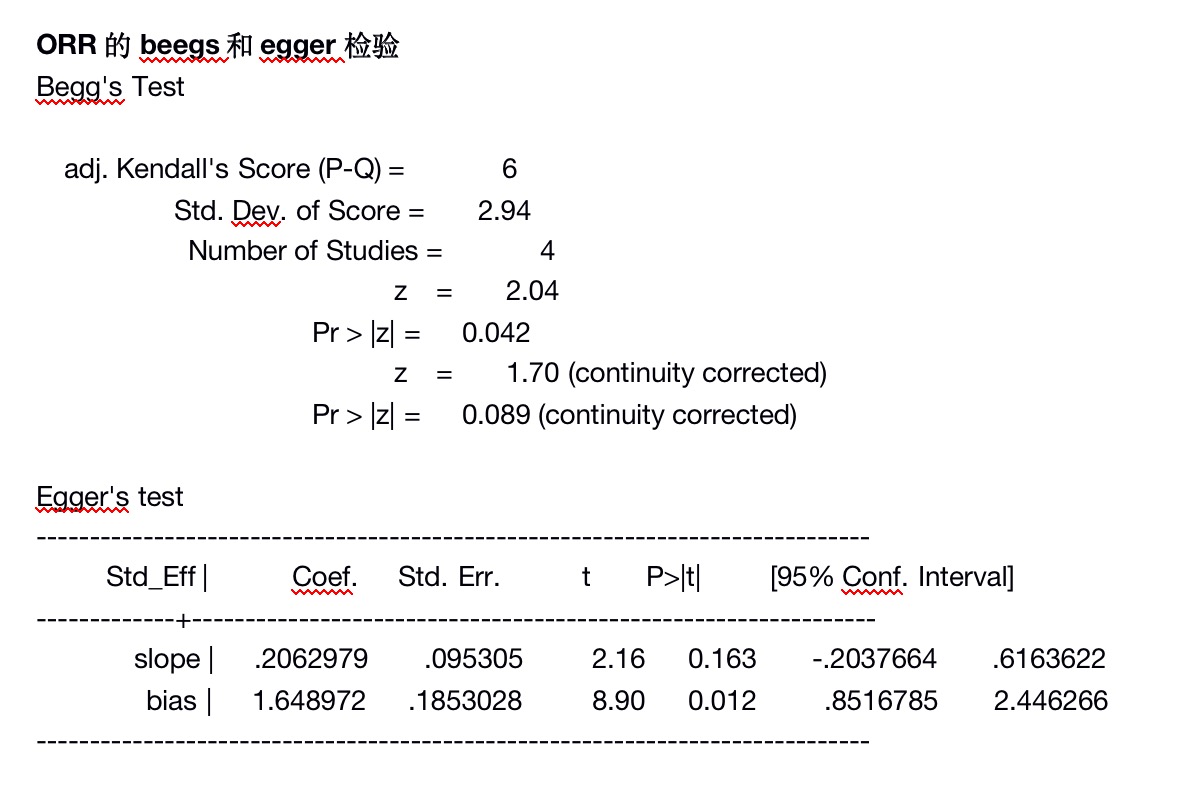
E


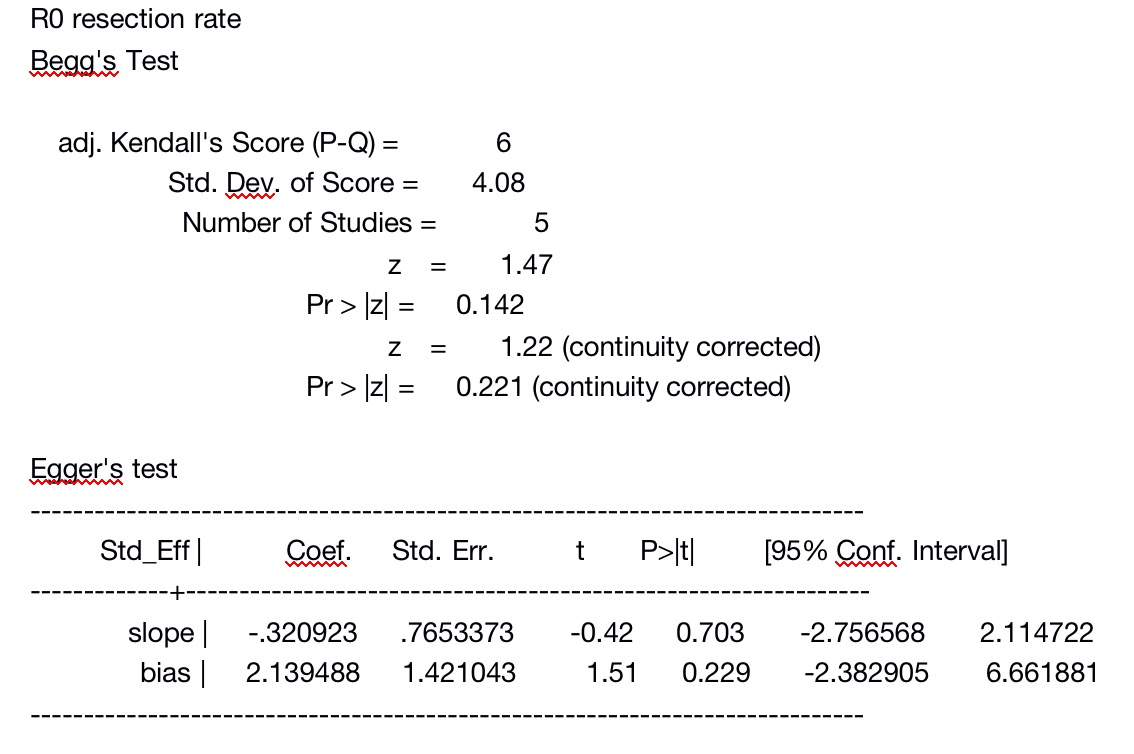


**
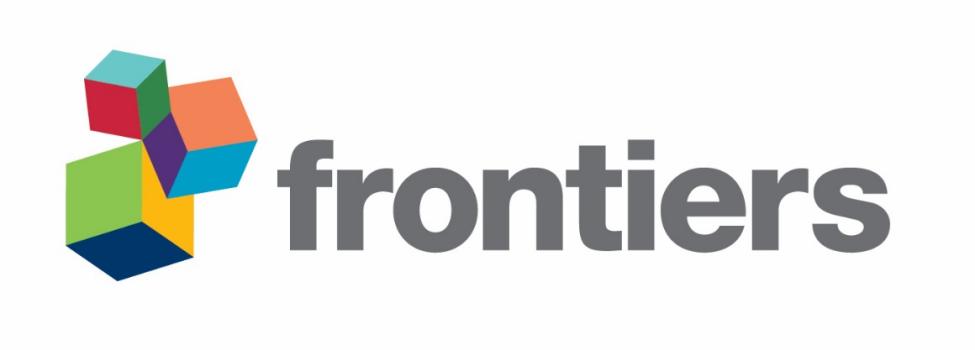
**
